# Bilateral–Contralateral Endoscopic Decompression as a Fusion-Deferral Strategy in Upper Lumbar Stenosis: A Structural Rationale and Conditional Framework—A Technical Note with Cases Review

**DOI:** 10.3390/jcm14165726

**Published:** 2025-08-13

**Authors:** Dong Hyun Lee, Sang Yeop Han, Seung Young Jeong, Il-Tae Jang

**Affiliations:** 1 Department of Neurosurgery, Spine Center, Nanoori Gangnam Hospital, Seoul 06048, Republic of Korea; 2 Department of Neurosurgery, Spine Center, Wiltse Memorial Hospital, Suwon 16480, Republic of Korea; 3 Department of Spine Center, Nanoori Juan Hospital, Incheon 22147, Republic of Korea

**Keywords:** bilateral–contralateral, upper lumbar stenosis, facet joint, unilateral biportal endoscopy (UBE), fusion deferral, adjacent segment disease (ASD), spondylolisthesis, endoscopic decompression

## Abstract

**Background/Objectives:** Upper lumbar spinal stenosis presents unique challenges because vertically oriented facet joints and narrow laminae increase the risk of iatrogenic instability following decompression. Traditional decompression techniques may damage the facet joints, potentially resulting in further instability and degeneration. This study introduces a novel, facet-preserving bilateral–contralateral decompression strategy using unilateral biportal endoscopy (UBE) for upper lumbar stenosis, aiming to defer unnecessary spinal fusion. **Methods:** This retrospective series of three cases involved patients with upper lumbar stenosis characterized by vertically oriented facets (>60°) and narrow laminae, including cases of adjacent segment stenosis (ASS) and stenosis with grade 1 spondylolisthesis. Patients were selected using the authors’ facet angle–based criteria (>60°) and laminar morphology to identify anatomically vulnerable segments. All patients exhibited vertical facet orientation and narrow laminae, without significant dynamic instability or severe foraminal compromise. Bilateral–contralateral decompression was performed using biportal endoscopy to preserve facet integrity and defer fusion where feasible. **Results:** This series demonstrated that bilateral–contralateral decompression provided effective neural decompression and symptom relief while preserving facet structures in the upper lumbar spine characterized by vertical facets and narrow laminae. No progression to instability or requirement for additional fusion was observed during the 6-month follow-up, even among patients with ASS and grade 1 spondylolisthesis. **Conclusions:** The authors propose that bilateral–contralateral decompression may serve as a facet-preserving and fusion-deferral strategy for upper lumbar stenosis with vertically oriented facets and narrow laminae. This approach is particularly applicable in cases such as ASS and spinal stenosis with grade 1 spondylolisthesis, where preserving structural reserve is critical. These preliminary findings highlight the need for prospective validation through carefully designed observational studies and larger case series.

## 1. Introduction

The anatomical definition of the upper lumbar spine remains somewhat controversial, particularly regarding whether the L3–L4 segment should be included [1,2]. Despite this, it is generally accepted that the L1–L3 region represents a unique zone where distinct morphological and biomechanical characteristics emerge. Various surgical techniques have been introduced for upper lumbar disc herniation, including transdural [3,4], oblique paraspinal [5], and transforaminal full-endoscopic approaches [6]. Recently, both the unilateral biportal endoscopic (UBE) method and upper lumbar approaches using O-arm navigation have been described [7,8,9].

Despite these advances, there remains a notable lack of literature focusing specifically on decompression strategies for upper lumbar stenosis. Although less common than lower lumbar disease, upper lumbar pathology presents unique anatomical challenges that impact surgical planning. The facet joints in the L1–L3 region tend to be more vertically oriented and structurally narrow, with a slender pars interarticularis, while the lamina is thinner and steeper than those in lower lumbar levels [10,11,12,13].

These anatomical features create a higher risk of iatrogenic damage during posterior decompression, especially with ipsilateral approaches that may partially resect the medial facet due to the narrow corridor and vertical orientation [12,14]. For upper lumbar stenosis, the optimal decompression technique remains less clearly defined.

Recent advances in minimally invasive spinal surgery—particularly unilateral biportal endoscopic (UBE) decompression targeting the pain-generating structures—have led to improved clinical outcomes and patient satisfaction [15,16,17,18,19,20]. Studies have shown that contralateral approaches preserve the facet joint better than ipsilateral approaches, especially when performed with endoscopic visualization [21,22,23,24].

**Figure 1:**
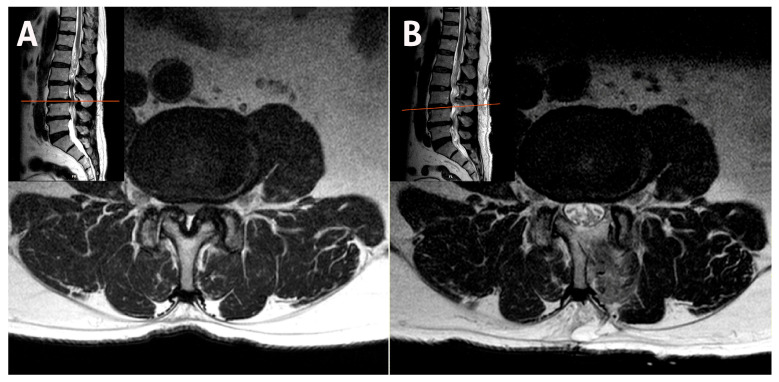
(**A**) MRI demonstrating spinal stenosis with vertically oriented facets at the L3–L4 level. (**B**) MRI following ipsilateral ULBD showing significant facet base resection in a patient with L3–L4 stenosis. MRI, magnetic resonance imaging; ULBD, unilateral laminotomy for bilateral decompression.

**Figure 2:**
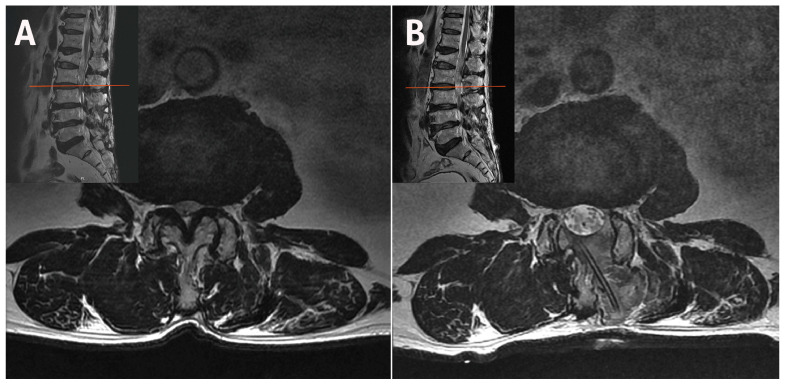
(**A**) MRI demonstrating spinal stenosis with vertically oriented facets at the L2–L3 level. (**B**) MRI showing loss of facet support after ipsilateral decompression at L2–L3. MRI, magnetic resonance imaging.

**Figure 3:**
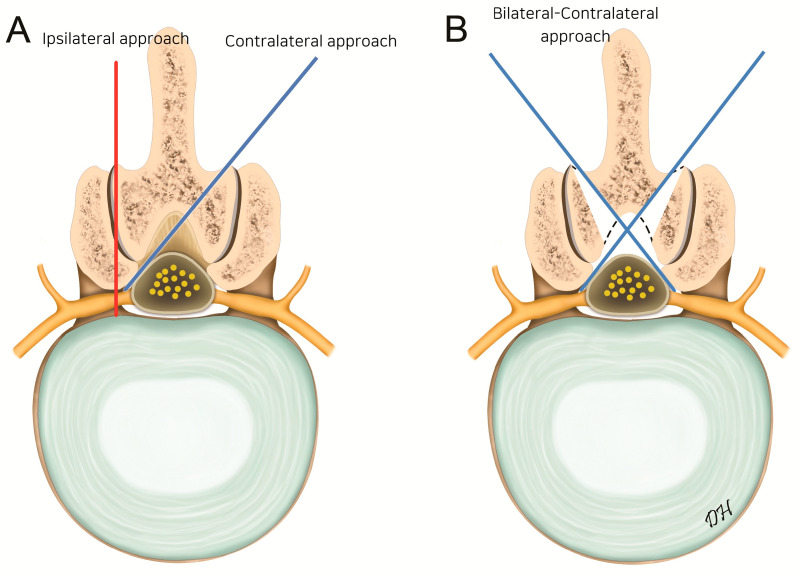
(**A**) Disparities in facet resection between the ipsilateral and contralateral methods in the upper lumbar. (**B**) Contralateral decompression was performed on both sides utilizing a unilateral biportal endoscopic technique employing a bilateral–contralateral approach.

The difference in facet preservation is particularly pronounced in the upper lumbar region, where vertically oriented facet joints and narrow laminae increase the risk that even minor over-resection may cause irreversible structural compromise. A magnetic resonance imaging (MRI) scan following ipsilateral unilateral laminotomy for bilateral decompression (ULBD) showed significant facet base resection in a patient with L3–L4 stenosis (Figure 1). If caution is not exercised, isthmus fractures can also occur, particularly in the upper lumbar region (Figure 2). A bilateral–contralateral approach was utilized [25,26], taking advantage of the contralateral pathway where the facet joints are minimally resected, as shown in Figure 3.

Early experiences indicate that this approach may be structurally protective. This study introduces a structured anatomical rationale for bilateral–contralateral decompression in upper lumbar pathology and offers practical guidance for selecting appropriate cases based on facet orientation and laminar geometry, as described in the following cases.

### Surgical Technique

**Figure 4:**
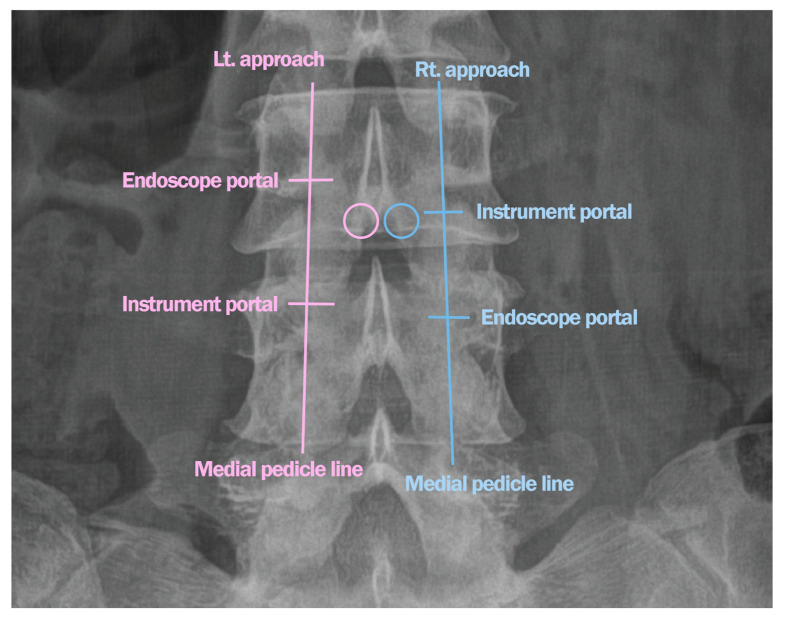
The circle indicates the initial targeting area: the spinolaminar junction. Skin incisions are made along the medial pedicle line, separated by 2–3 cm. Lt., left; Rt., right.

Bilateral–contralateral UBE decompression was performed under general anesthesia with the patient prone on a radiolucent Relton–Hall frame. Two incisions were made on each side along the medial pedicle line (Figure 4). The left-sided approach involved cranial lamina drilling and contralateral thinning, preserving the ligamentum flavum as a barrier. In severe stenosis, partial ipsilateral outer-flavum resection was necessary to visualize the contralateral side.

After decompressing the contralateral traversing nerve root by flavum removal and facet undercutting, the surgeon reentered from the right side via a mirrored portal.

**Figure 5:**
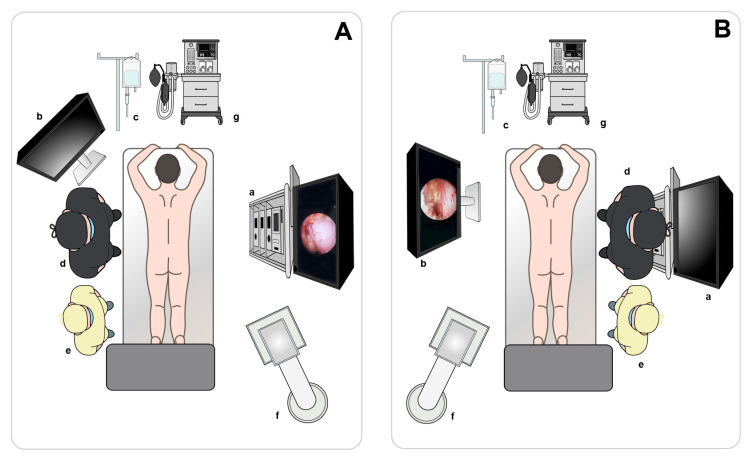
(**A**) Accessing from the left to decompress the right side. (**B**) Accessing from the right to decompress the left side. Dual monitors enable immediate transition to the opposite side for seamless decompression. The surgical set-up consists of: (a) endoscopy tower; (b) dual monitor; (c) saline bag; (d) surgeon; (e) scrub nurse; (f) C-arm; and (g) anesthesia machine.

Twin monitors enabled the surgeon to switch to the opposite side and immediately begin the contralateral approach without moving the UBE tower (Figure 5). The right incision was made approximately 5–10 mm lower than the left to avoid excessive laminectomy caused by hand angulation (Figure 4). The sublaminar plane above the contralateral ligamentum flavum was reached through the prior decompression site with minimal additional laminectomy. Laminectomy was performed only on the portion of the right lamina that interfered with entry into the epidural space, and contralateral decompression was conducted using the same technique.

Radiofrequency coagulation ensured hemostasis. A continuous saline flow helped prevent heat damage and maintain visibility. Full technical details have been described in previous publications [25,26].

## 2. Materials and Methods

### 2.1. Case 1: Bilateral–Contralateral Decompression in Upper Lumbar Stenosis

**Figure 6:**
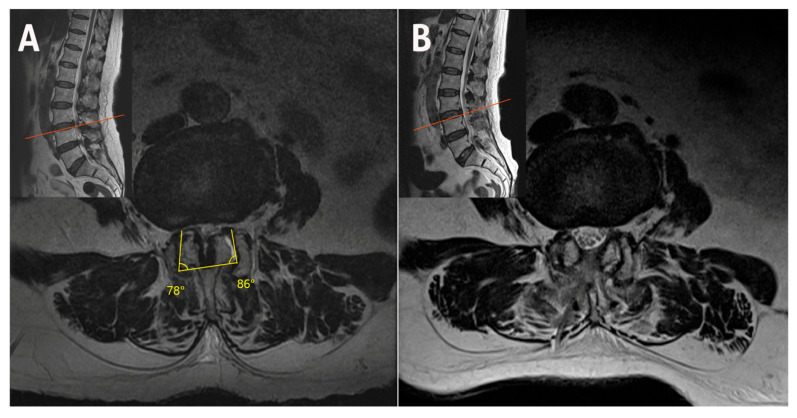
(**A**) Facet angles were measured as the angle between a line connecting the two ends of the facet joint surface and a reference line along the posterior margin. The average value (82.0 degrees in this case) was utilized when the facet angles differed (right side vs. left side). (**B**) Postoperative MRI after bilateral–contralateral decompression at L3–L4 showing preserved bilateral facet bases in vertically oriented joints. MRI, magnetic resonance imaging.

A 67-year-old male patient with L3–L4 stenosis and vertically oriented facets underwent bilateral–contralateral decompression using a biportal endoscopic approach. Stability was assessed preoperatively using flexion–extension radiographs, with dynamic instability defined as >3 mm translation or >10° angular motion. Postoperative MRI was performed to confirm adequate decompression and facet joint preservation. This protocol was applied consistently to all cases. Postoperative imaging demonstrated preservation of bilateral facet bases and lateral recess decompression without compromising stability. This case represents a typical example of upper lumbar stenosis, where the structural preservation advantage of the bilateral–contralateral technique is most evident (Figure 6).

The postoperative MRI scan following bilateral–contralateral decompression at L3–L4 shows preserved bilateral facet bases in vertically oriented joints. The operative time for this case was 55 min.

### 2.2. Case 2: Bilateral–Contralateral Decompression in Adjacent Segmental Stenosis (ASS) After Lumbar Fusion

**Figure 7:**
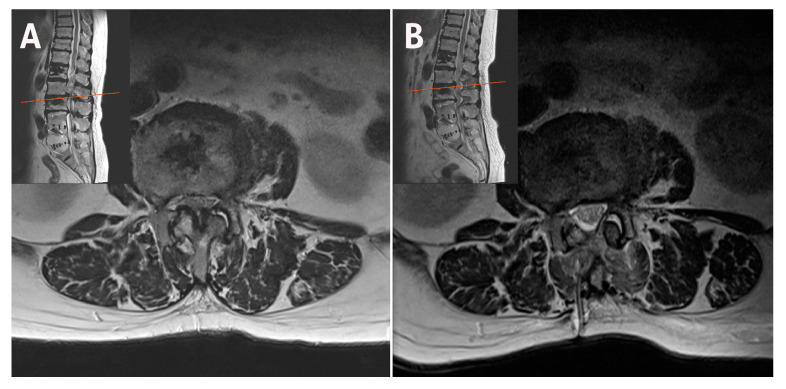
(**A**) MRI demonstrating vertically oriented facets at the L2–L3 level associated with ASS. (**B**) Postoperative MRI showing decompressed L2–L3 recesses in a patient with ASS and preserved orientation of both facets. MRI, magnetic resonance imaging; ASS, adjacent segment stenosis.

An 82-year-old female patient with prior L4–L5-S1 fusion presented with adjacent segmental stenosis at L2–L3 with a vertical facet angle. Bilateral–contralateral decompression was performed, achieving full neural decompression and preservation of facet integrity on postoperative imaging (Figure 7). The operative time was 68 min.

### 2.3. Case 3: Bilateral–Contralateral Decompression in Spondylolisthesis

**Figure 8:**
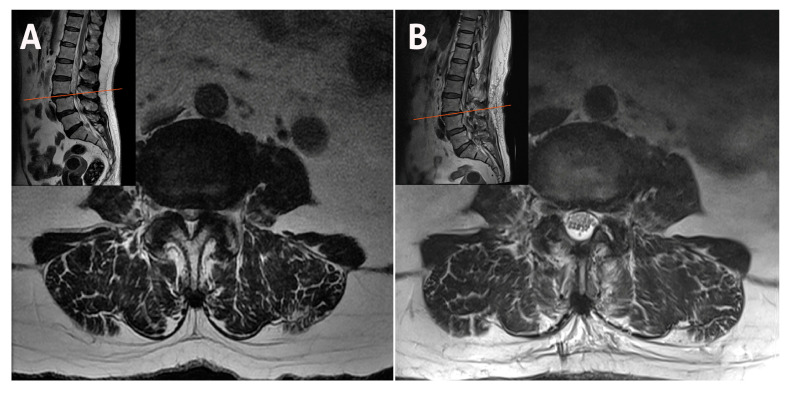
(**A**) MRI demonstrating spinal stenosis with vertically oriented facets at the L3–L4 level with grade 1 spondylolisthesis. (**B**) MRI image of a patient with spondylolisthesis post-decompression showing preservation of both facets. MRI, magnetic resonance imaging.

A 78-year-old female patient with L3–L4 stenosis with grade 1 spondylolisthesis underwent bilateral–contralateral decompression using the same technique. This approach enabled undercutting decompression of the lateral recess while preserving the integrity of the medial facet base. Both facets were preserved, and no instability was observed on follow-up imaging (Figure 8). The operative time was 64 min.

These cases illustrate that bilateral–contralateral endoscopic decompression can achieve decompression goals while preserving facet structures in biomechanically vulnerable settings. Table 1 summarizes the demographics, operative details, and clinical outcomes of the three illustrative cases. This consideration may be particularly relevant in the upper lumbar region, where the facet joints are more vertically oriented and tolerance for iatrogenic damage is lower. In selected cases, including ASS and spinal stenosis with spondylolisthesis, where structural reserve is already compromised in the upper lumbar spine, this technique may offer an alternative to more invasive fusion strategies.

## 3. Discussion

Various surgical strategies have been explored for upper lumbar disc herniation. Traditional transdural techniques, though facet-preserving, require both dorsal and ventral durotomies, elevating the risk of cerebrospinal fluid leak and neural injury [3,4]. Kim et al. [5] described the development of an oblique paraspinal microscopic approach that targets only the anterolateral facet and lateral pars. Endoscopic techniques—especially transforaminal approaches such as transforaminal endoscopic lumbar discectomy [6,27]—have shown promise, including a recent biportal UBE adaptation specifically applied to upper lumbar disc pathology [8,28,29].

Anatomically, the upper lumbar region presents narrower laminae and vertically oriented facet joints, increasing the risk of iatrogenic destabilization. However, despite these anatomical challenges, no decompression-specific strategy has been systematically applied to upper lumbar stenosis, particularly in patients for whom preserving structural integrity is critical.

Our approach builds upon this anatomical understanding while addressing the limitations of existing ipsilateral decompression in vertically aligned facet joints.

Figure 6 demonstrates decompression with minimal damage to the stabilizing structures in the narrow laminae and vertical facets of the upper lumbar region.

Although the evidence is mainly derived from a cadaveric study, facet resection may appear to be compensated for by alternative load paths under normal conditions [30]. However, these findings do not fully reflect the compromised reserve in upper lumbar stenosis, particularly in ASD or spondylolisthesis settings where facet preservation can help defer unnecessary fusion. Given the vertical facet orientation and limited structural reserve in the upper lumbar region, even partial resection may destabilize the segment. Therefore, bilateral–contralateral techniques should not be applied universally to stable upper lumbar pathology but may be considered selectively in patients with compromised segments, especially when the goal is to minimize the possibility of instability in elderly patients with significant comorbidities who cannot undergo fusion surgery. These considerations support the need for a more structure-preserving decompression strategy.

Since fusion is generally considered the standard treatment for ASD, the indication for stand-alone decompression in this context remains controversial [31,32,33,34]. Although a full discussion of these indications is beyond the scope of this paper, Drysch et al. [35] proposed a treatment-based categorization scheme for adjacent segment degeneration based on the presence of instability and stenosis. Based on their framework, when instability is evident in nearby segments—regardless of stenosis—decompression and fusion may be more appropriate. Conversely, if stenosis is present and the spine is stable, a laminectomy can suffice.

When an adjacent segment laminectomy is required, performing surgery that preserves the posterior complex offers the advantage of preventing future degeneration [36,37,38].

Additionally, in cases vulnerable to instability, such as ASD following decompression—particularly when the lamina is narrow and the facet angle exceeds 60 degrees, or even 70 degrees in the upper lumbar spine—bilateral–contralateral decompression using the bi-contra technique has been reported to yield stable outcomes while preserving both facets [25]. Wang and Green [39] showed that lumbar decompression can be safely performed in elderly patients; however, prolonged operative time, such as that required for fusion extension, was associated with a higher complication rate. This finding underscores the importance of minimizing unnecessary surgical extensions in frail patients when feasible.

The bilateral–contralateral decompression technique in the upper lumbar spine, characterized by a vertical facet orientation, offers the advantage of preserving both facets, which could be critical for addressing ASS, preventing post-decompression instability, slowing ASD progression, and providing a pragmatic fusion-deferral option.

This bilateral–contralateral decompression method can be selectively applied to patients with spinal stenosis and low-grade spondylolisthesis, in whom residual instability remains a clinical concern. The technique was initially developed for cases involving stenosis with spondylolisthesis [26], including segments in the upper lumbar spine where vertical facet orientation and a narrow lamina reduce structural reserve.

It is generally accepted that lumbar spine instability is caused by mobile degenerative spondylolisthesis with mechanical low back pain, and that the recommended treatment is decompression with fusion [40,41]. Other studies have reported comparable outcomes with decompression alone in carefully selected stable cases [42,43,44,45].

Nonetheless, the risk of post-decompression instability persists, especially in anatomically vulnerable segments of the upper lumbar spine with vertical facets and a narrow pars interarticularis, as supported by recent morphometric and biomechanical studies [10,12,46,47,48]. Biomechanical studies have demonstrated that even partial medial facetectomy can significantly reduce segmental stiffness, particularly in the upper lumbar spine, where facet orientation plays a more direct stabilizing role [38]. These findings support the anatomical basis for facet preservation when appropriate, especially when using bilateral–contralateral approaches to minimize facet violation and maintain posterior structural integrity.

This approach may be particularly beneficial in elderly or medically frail patients for whom fusion poses heightened risks, providing a pragmatic alternative in fusion-limited scenarios where facet-preserving decompression meets a distinct clinical need.

Historically, upper lumbar decompression strategies have lacked a nuanced anatomical rationale. These limitations can be attributed to (1) the dominance of fusion-based treatment paradigms, (2) limited technical means for anatomical preservation in pre-endoscopic eras, and (3) the infrequent and often unsegmented reporting of upper lumbar cases. With the advent of biportal endoscopy, safer contralateral approaches now enable anatomy-based fusion deferral via stability-preserving decompression, supporting a reevaluation of decompression strategies in this anatomically distinct region.

The concept of bilateral–contralateral decompression was first introduced in endoscopic spine surgery by Lee et al. [26], and its application to ASS was further elaborated in subsequent research [25]. While microscopic bilateral-crossing techniques have been described historically [49], their scope of application and indications in endoscopic bilateral–contralateral decompression—particularly for upper lumbar pathology—were subsequently refined and systematically formalized through our reports.

The bilateral–contralateral approach should be used with caution following rigorous selection of patients with minimal sagittal motion. In our previous dataset, factors such as high facet angle (>60°), spondylolisthesis, and segmental mobility [25,26] were not statistically associated with postoperative instability. While this may reflect limitations in sample size or study design, it may also suggest that the bilateral–contralateral approach is less susceptible to these anatomical risk factors, thereby supporting its potential structural reliability.

As the population continues to age, the importance of endoscopic treatment is increasingly recognized. We emphasize that the bilateral–contralateral technique is not universally required but may serve as a structure-preserving option in cases of specific anatomical vulnerabilities.

**Figure 9:**
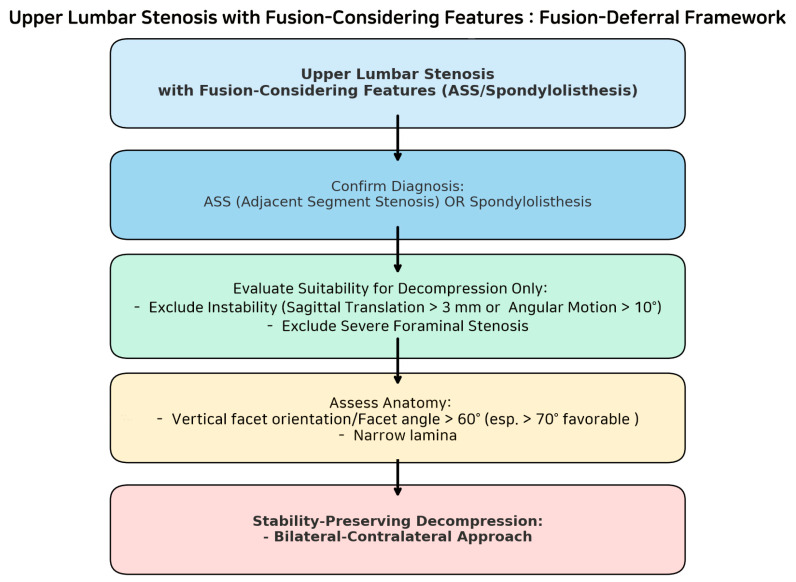
DH criteria flowchart for patient selection in bilateral–contralateral decompression for upper lumbar stenosis with fusion-considering features.

For older patients with multiple comorbidities, the authors’ (DH, Lee) criteria suggest considering bilateral–contralateral decompression at the upper lumbar spine—particularly when facet joint angles exceed 60 degrees in cases of ASS or spinal stenosis with spondylolisthesis (Figure 9). This approach minimizes facet joint resection and may prevent delayed progressive instability, potentially averting the need for additional fixation surgery in medically frail patients, for whom reoperation or extended fusion poses heightened risks.

This strategy may serve as a pragmatic alternative in fusion-limited scenarios, addressing a distinct clinical need while potentially reducing unnecessary fusion in elderly or comorbid patients, although formal cost-effectiveness analyses remain necessary.

Because UBE involves the dissection of the multifidus muscles on both sides, the bilateral–contralateral approach is less minimally invasive regarding muscle preservation than standard unilateral methods. However, previous studies on UBE have shown minimal multifidus injury and a return to preoperative muscle status within a few months, indicating that this limitation may be transient and clinically acceptable [50].

While prospective randomized controlled trials (RCTs) may offer additional validation in the future, the unique nature of this technique—marked by high operator-dependency, anatomical variability, and nuanced patient selection—poses inherent limitations to conventional RCT designs.

In such contexts, repetitive observational research guided by clearly theorized clinical criteria—such as the DH criteria—may provide a more appropriate and robust framework for advancing facet-preserving decompression strategies [51,52,53,54,55].

These informal working criteria, based on recurring patterns observed in our prior cases, offer practical guidance in identifying surgical candidates with upper lumbar vulnerability and warrant further evaluation in both retrospective and prospective settings to assess their long-term effect on fusion deferral and degeneration suppression in these targeted subgroups.

## 4. Limitations

This series is observational and lacks a randomized control group. While no instability was observed on follow-up imaging, long-term durability remains unproven. Furthermore, selection criteria—such as the absence of foraminal stenosis and dynamic instability—are key and may limit broader applicability. Additional prospective or biomechanical studies are warranted. Anatomy-informed surgical strategies often elude rigid RCT frameworks; thus, additional registry-based and prospective observational studies remain the most practical means of validating fusion deferral effects in these specific subgroups. Importantly, the current short-term findings are contextualized by previously published 1- and ≥2-year follow-up series [25,26], which demonstrate the feasibility and sustained stability of this technique in selected patients.

## Figures and Tables

**Table 1:** Patient demographics and clinical outcomes.

Case	Age (years)	Sex	Diagnosis	Level	Operative Time (min)	Pre-op Back VAS	Post-op Back VAS	Pre-op Leg VAS	Post-op Leg VAS	Pre-op ODI	Post-op ODI	Complications	Follow-Up (Months)
1	67	M	Upper Lumbar Stenosis	L3–L4	55	6	2	7	1	48	16	None	6
2	82	F	Adjacent Segment Stenosis	L2–L3	68	7	3	8	3	58	26	None	6
3	78	F	Stenosis with Spondylolisthesis	L3–L4	64	6	2	8	2	50	20	None	6

VAS, Visual Analog Scale; ODI, Oswestry Disability Index.

## Data Availability

Data are available from the corresponding author upon reasonable request.

## Abbreviations

The following abbreviations are used in this manuscript:

ASS	Adjacent segment stenosis
ASD	Adjacent segment disease
MRI	Magnetic resonance imaging
RCT	Randomized controlled trial
UBE	Unilateral biportal endoscopy
ULBD	Unilateral laminotomy for bilateral decompression
